# Supplementary figures and images for: Phylogeography and population genetic structure of the cardinal tetra (Paracheirodon axelrodi) in the Orinoco basin and Negro River (Amazon basin): evaluating connectivity and historical patterns of diversification
Source: PeerJ. 2023 Jun 8;11:e15117. doi: 10.7717/peerj.15117 (PMC10257900; doi:10.7717/peerj.15117)

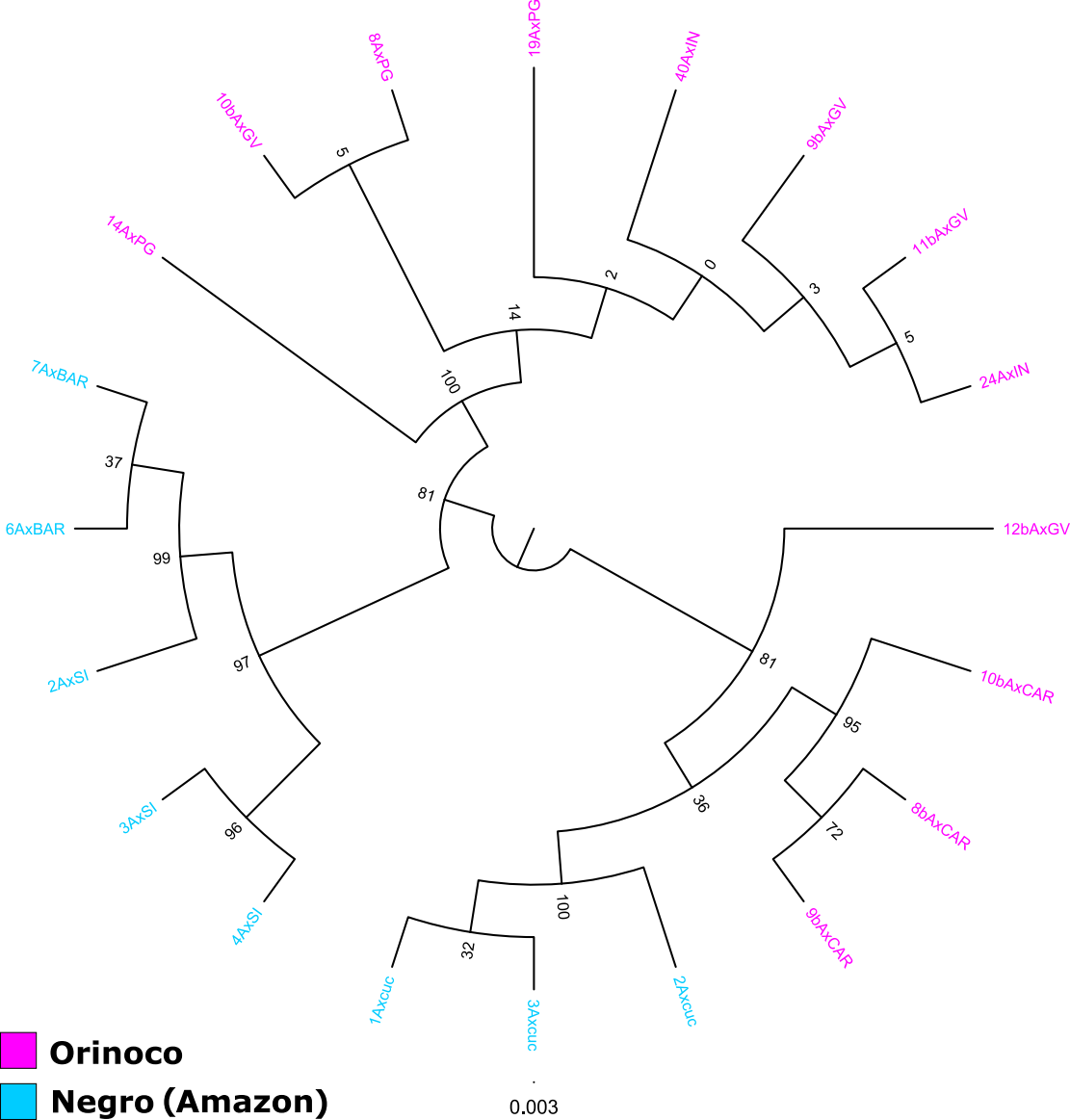

Supplement: Supplemental Information 1 [file peerj-11-15117-s001.pdf]

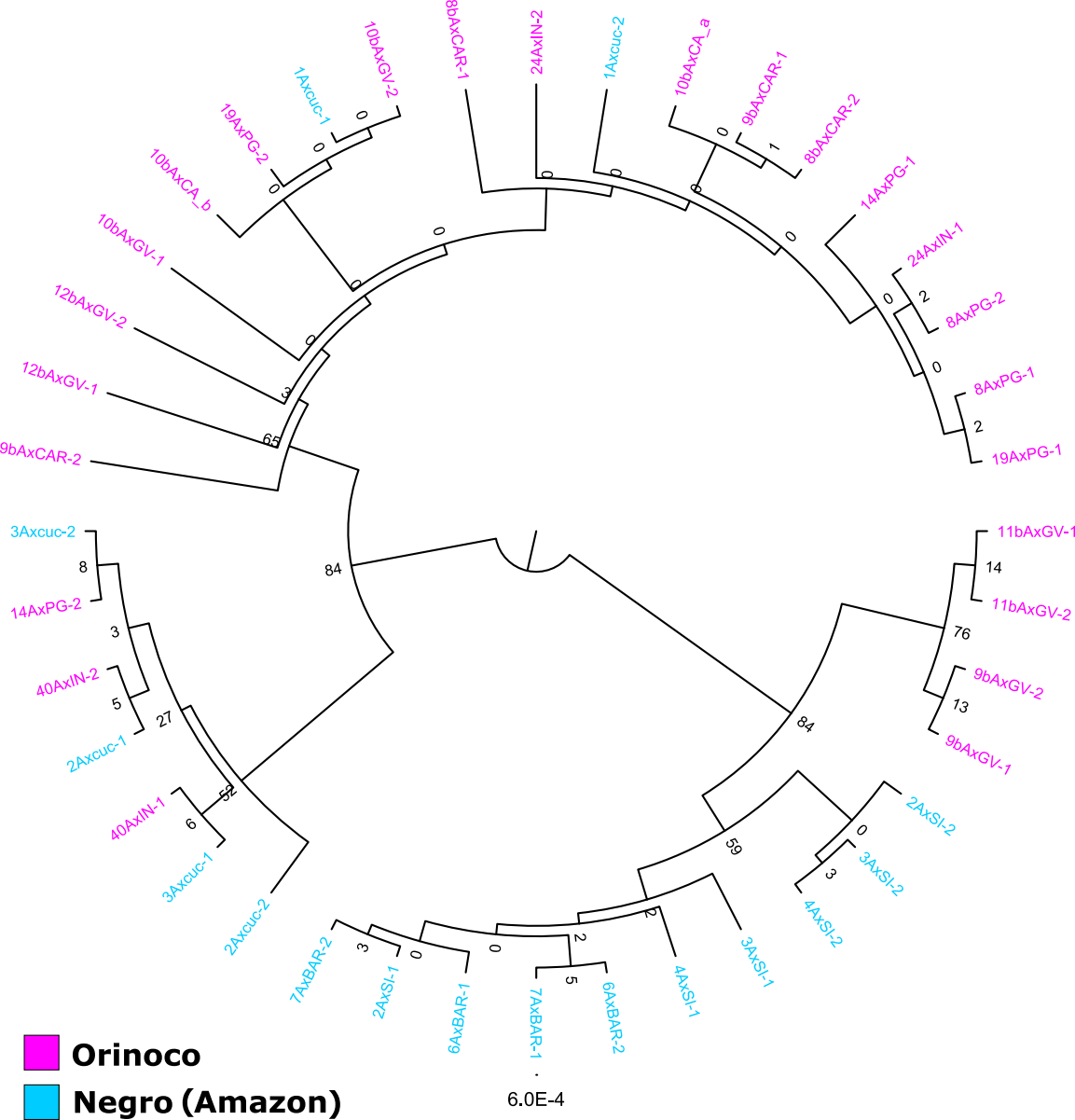

Supplement: Supplemental Information 2 [file peerj-11-15117-s002.pdf]

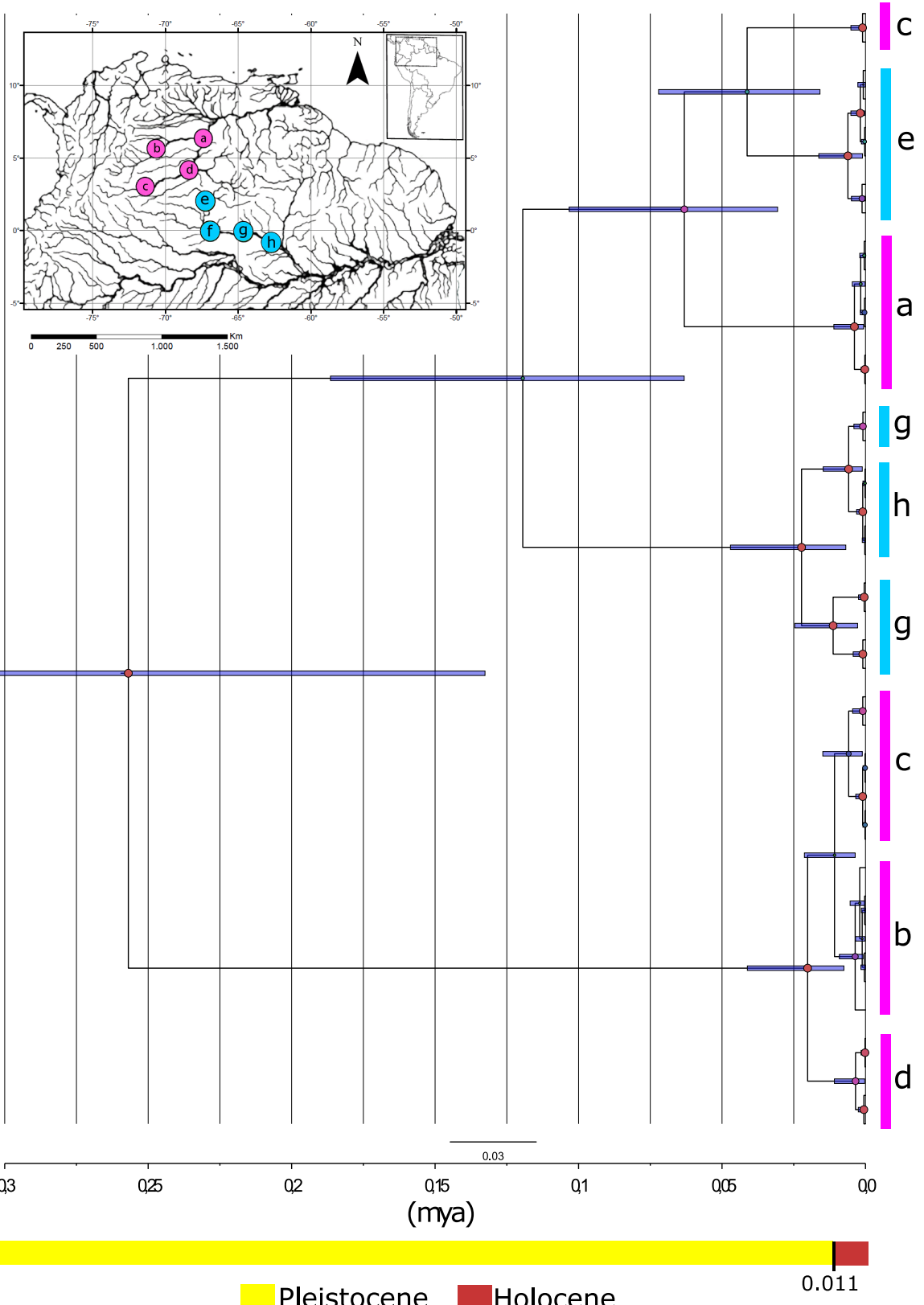

Supplement: Supplemental Information 3 — Samples from Orinoco River basin ((A) Puerto Carreño; (B) Puerto Gaitán; (C) San José del Guaviare; (D) Inírida) and Negro River basin ((E) Cucuí; (F) São Gabriel da Cachoeira; (G) Santa Isabel; (H) Barcelos), using COI and MYH6 exonic region, estimated under a HKY+GAMMA and GTR+GAMMA models of molecular evolution, respectively. The observed times are median heights into 95% credibility intervals, calibrated with the widely accepted mtDNA substitution rate for poikilotherm vertebrates in Martin & Palumbi (1993) of 0.68 ×10^-8^ mutations per site per year for COI, and 1.0 ×10^-9^ for MYH6 (Freeland, 2005). Circles indicate nodes with supports ≥ 0.95 of posterior probability. [file peerj-11-15117-s003.pdf]

**A****Delta K**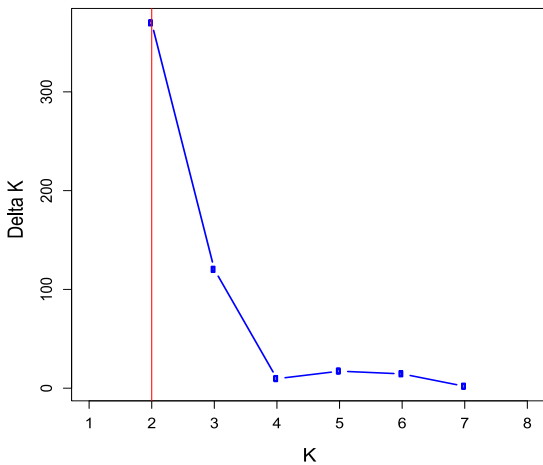**B****MedMed K**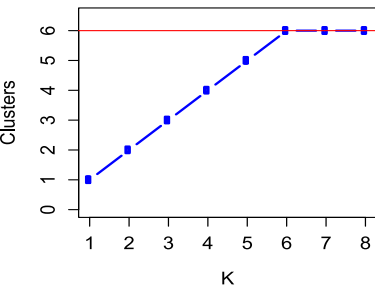**MedMean K**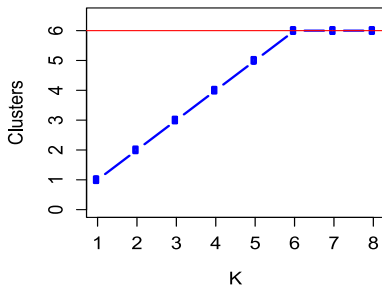**MaxMed K**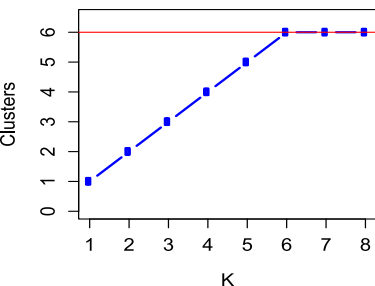**MaxMean K**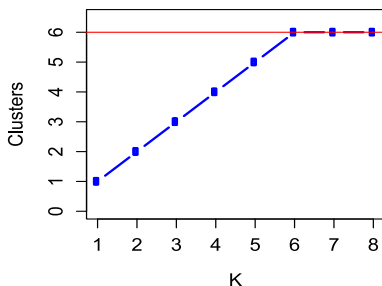

Supplement: Supplemental Information 4 — (A) Evanno method (Δ K), identifying K = 2 as the most likely number of clusters. (C) Puechmille method estimators (MaxMed and MaxMean) showing K = 6 as the most likely number of clusters. [file peerj-11-15117-s004.pdf]
