# Supplementary material for: Phylogeography and population genetic structure of the cardinal tetra (Paracheirodon axelrodi) in the Orinoco basin and Negro River (Amazon basin): evaluating connectivity and historical patterns of diversification
Source: PeerJ. 2023 Jun 8;11:e15117. doi: 10.7717/peerj.15117 (PMC10257900; doi:10.7717/peerj.15117)
Supplement: Supplemental Information 9 — N = Cardinal sample size; for each locus: n = total number of alleles, Ho = observed heterozygosity, HE = expected heterozygosity. Loci out of equilibrium after Bonferroni correction (0.00078125) are shown in bold. CAR: Puerto Carreño; GV: San José del Guaviare; IN: Puerto Inírida; PG: Puerto Gaitán; CUC: Cucuí; SI: Santa Isabel; BAR: Barcelos. [file peerj-11-15117-s009.docx]

**Table S2.** Genetic diversity for eight microsatellite loci in all *P. axelrodi* geographic locations analyzed. *N=* Cardinal sample size; for each locus: *n= total* number of alleles, *Ho*= observed heterozygosity, *HE*= expected heterozygosity. Loci out of equilibrium after Bonferroni correction (0.00078125) are shown in bold. CAR: Puerto Carreño; GV: San José del Guaviare; IN: Puerto Inirida; PG: Puerto Gaitan; CUC: Cucui; SI: Santa Isabel; BAR: Barcelos.

| **Locus** | **GV** | **CAR** | **PG** | **IN** | **CUC** | **BAR** | **SI** | **SGC** |
| --- | --- | --- | --- | --- | --- | --- | --- | --- |
|  | N=20 | N=20 | N=20 | N=20 | N=21 | N=20 | N=20 | N=20 |
| **Pa7** | *n=* 6 | *n=* 4 | *n=* 7 | *n=* 5 | *n=* 10 | *n=* 9 | *n=* 7 | *n=* 8 |
|  | *Ho*= 0.80 | *Ho*= 0.60 | *Ho*= 0.71 | *Ho*= 0.76 | *Ho*= 1.00 | *Ho*= 0.90 | *Ho*= 0.90 | *Ho*= 0.50 |
|  | *HE* = 0.74 | *HE* = 0.62 | *HE* = 0.75 | *HE* = 0.60 | *HE* = 0.89 | *HE* = 0.69 | *HE* = 0.78 | *HE* = 0.67 |
|  | *P=* 0.009 | *P=* 0.164 | *P=* 0.062 | *P=* 0.458 | *P=* 0.411 | *P=* 0.040 | *P=* 0.669 | *P=* 0.132 |
|  |  |  |  |  |  |  |  |  |
| **Pa13** | *n=* 3 | ***n=* 8** | *n=* 6 | *n=* 6 | *n=* 8 | *n=* 5 | *n=* 7 | *n=* 6 |
|  | *Ho*= 0.40 | ***Ho*= 0.42** | *Ho*= 0.43 | *Ho*= 0.61 | *Ho*= 0.80 | *Ho*= 0.65 | *Ho*= 0.80 | *Ho*= 0.63 |
|  | *HE*= 0.48 | ***HE*= 0.75** | *HE*= 0.57 | *HE* = 0.72 | *HE*= 0.81 | *HE*= 0.64 | *HE*= 0.77 | *HE*= 0.74 |
|  | *P=* 1.000 | ***P=* 0.000** | *P=* 0.106 | *P=* 0.145 | *P=* 0.370 | *P=* 0.127 | *P=* 0.064 | *P=* 0.089 |
|  |  |  |  |  |  |  |  |  |
| **Pa19** | *n=* 5 | *n=* 6 | *n=* 2 | ***n=* 5** | *n=* 5 | *n=* 7 | *n=* 10 | ***n=* 5** |
|  | *Ho*= 0.50 | *Ho*= 0.50 | *Ho*= 0.11 | ***Ho*= 0.10** | *Ho*= 0.72 | *Ho*= 0.75 | *Ho*= 0.63 | ***Ho*= 0.20** |
|  | *HE*= 0.56 | *HE* = 0.78 | *HE*= 0.10 | ***HE* = 0.55** | *HE*= 0.64 | *HE*= 0.81 | *HE*= 0.88 | ***HE*= 0.80** |
|  | *P=* 0.196 | *P=* 0.006 | *P=* 1.000 | ***P=* 0.000** | *P=* 0.315 | *P=* 0.144 | *P=* 0.023 | ***P=* 0.000** |
|  |  |  |  |  |  |  |  |  |
| **Pa26** | n=11 | *n=* 7 | ***n=* 7** | *n=* 7 | *n=* 6 | *n=* 2 | *n=* 10 | *n=* 5 |
|  | *Ho*= 0.60 | *Ho*= 0.65 | ***Ho*= 0.55** | *Ho*= 0.47 | *Ho*= 0.65 | *Ho*= 0.15 | *Ho*= 0.55 | *Ho*= 0.70 |
|  | *HE* = 0.82 | *HE* = 0.58 | ***HE*= 0.66** | *HE* = 0.63 | *HE* = 0.72 | *HE* = 0.45 | *HE* = 0.69 | *HE* = 0.73 |
|  | *P=* 0.005 | *P=* 0.368 | ***P=* 0.000** | *P=* 0.101 | *P=* 0.622 | *P=* 0.004 | *P=* 0.027 | *P=* 0.025 |
|  |  |  |  |  |  |  |  |  |
| **Pa27** | **n=6** | n=3 | n=8 | **n=8** | n=5 | n=3 | n=7 | **n=6** |
|  | ***Ho*= 0.30** | *Ho*= 0.15 | *Ho*= 0.52 | ***Ho*= 0.38** | *Ho*= 0.71 | *Ho*= 0.10 | *Ho*= 0.61 | ***Ho*= 0.35** |
|  | ***HE*= 0.52** | *HE* = 0.14 | *HE*= 0.78 | ***HE* = 0.64** | *HE* = 0.72 | *HE* = 0.14 | *HE* = 0.64 | ***HE*= 0.76** |
|  | ***P=* 0.0007** | *P=* 1.000 | *P=* 0.013 | ***P=* 0.0006** | *P=* 0.012 | *P=* 0.076 | *P=* 0.372 | ***P=* 0.0001** |
|  |  |  |  |  |  |  |  |  |
|  |  |  |  |  |  |  |  |  |
| **Pa32** | **n=18** | **n=12** | **n=11** | **n=22** | **n=13** | **n=18** | **n=15** | **n=9** |
|  | ***Ho*= 0.52** | ***Ho*= 0.31** | ***Ho*= 0.45** | ***Ho*= 0.52** | ***Ho*= 0.50** | ***Ho*= 0.55** | ***Ho*= 0.39** | ***Ho*= 0.10** |
|  | ***HE*= 0.93** | ***HE* = 0.91** | ***HE*= 0.81** | ***HE* = 0.96** | ***HE* = 0.93** | ***HE* = 0.93** | ***HE* = 0.89** | ***HE*= 0.91** |
|  | ***P=* 0.0000** | ***P=* 0.0000** | ***P=* 0.00007** | ***P=* 0.0000** | ***P=* 0.0000** | ***P=* 0.0000** | ***P=* 0.0000** | ***P=* 0.0000** |
|  |  |  |  |  |  |  |  |  |
| **Pa33** | n=14 | **n=11** | n=12 | **n=10** | n=11 | n=4 | **n=7** | n=8 |
|  | *Ho*= 0.60 | ***Ho*= 0.55** | *Ho*= 0.71 | ***Ho*= 0.61** | *Ho*= 0.80 | *Ho*= 0.45 | ***Ho*= 0.45** | *Ho*= 0.50 |
|  | *HE*= 0.84 | ***HE*= 0.89** | *HE*= 0.82 | ***HE* = 0.86** | *HE* = 0.83 | *HE* = 0.66 | ***HE* = 0.77** | *HE*= 0.77 |
|  | *P=* 0.0014 | ***P=* 0.0003** | *P=* 0.346 | ***P=* 0.0052** | *P=* 0.036 | *P=* 0.0049 | ***P=* 0.0000** | *P=* 0.0072 |
|  |  |  |  |  |  |  |  |  |
| **Pa37** | n=6 | n=10 | **n=15** | n=17 | n=11 | **n=11** | n=14 | n=15 |
|  | *Ho*= 0.29 | *Ho*= 0.55 | ***Ho*= 0.50** | *Ho*= 0.76 | *Ho*= 0.66 | ***Ho*= 0.55** | *Ho*= 0.65 | *Ho*= 0.68 |
|  | *HE*= 0.49 | *HE* = 0.70 | ***HE*= 0.87** | *HE*= 0.90 | *HE*= 0.84 | ***HE*= 0.88** | *HE*= 0.92 | *HE*= 0.91 |
|  | *P=* 0.0042 | *P=* 0.033 | ***P=* 0.0005** | *P=* 0.0099 | *P=* 0.1103 | ***P=* 0.0003** | *P=* 0.0024 | *P=* 0.0047 |
