# Supplementary material for: Phylogeography and population genetic structure of the cardinal tetra (Paracheirodon axelrodi) in the Orinoco basin and Negro River (Amazon basin): evaluating connectivity and historical patterns of diversification
Source: PeerJ. 2023 Jun 8;11:e15117. doi: 10.7717/peerj.15117 (PMC10257900; doi:10.7717/peerj.15117)
Supplement: Supplemental Information 10 — Pairwise FST values using eight microsatellites loci among Orinoco and Negro basin populations for Paracheirodon axelrodi. Significantly different values (P < 0.05) are shown in bold. CAR: Puerto Carreño; GV: San José del Guaviare; IN: Puerto Inírida; PG: Puerto Gaitan; CUC: Cucui; SGC: São Gabriel da Cachoeira; SI: Santa Isabel; BAR: Barcelos. [file peerj-11-15117-s010.docx]

**Table S3.** Pairwise *F_ST_* values using eight microsatellites loci among Orinoco and Negro basin populations. Significantly different values (P < 0.05) are shown in bold. CAR: Puerto Carreño; GV: San José del Guaviare; IN: Puerto Inirida; PG: Puerto Gaitan; CUC: Cucui; SGC: Sao Gabriel da Cachoeira; SI: Santa Isabel; BAR: Barcelos.

|  | **GV** | **CAR** | **PG** | **IN** | **CUC** | **BAR** | **SI** | **SGC** |
| --- | --- | --- | --- | --- | --- | --- | --- | --- |
| *F_ST_* |  |  |  |  |  |  |  |  |
| **GV** |  |  |  |  |  |  |  |  |
| **CAR** | **0.24256** |  |  |  |  |  |  |  |
| **PG** | **0.15438** | **0.12465** |  |  |  |  |  |  |
| **IN** | **0.10255** | **0.12036** | 0.01036 |  |  |  |  |  |
| **CUC** | **0.11032** | **0.16970** | **0.09452** | **0.08932** |  |  |  |  |
| **BAR** | **0.31446** | **0.19739** | **0.18891** | **0.22822** | **0.17551** |  |  |  |
| **SI** | **0.20821** | **0.16410** | **0.12449** | **0.14715** | **0.09498** | **0.05115** |  |  |
| **SGC** | **0.15866** | **0.12738** | **0.05435** | **0.06491** | **0.06192** | **0.09914** | **0.03683** |  |
