# Supplementary material for: Phylogeography and population genetic structure of the cardinal tetra (Paracheirodon axelrodi) in the Orinoco basin and Negro River (Amazon basin): evaluating connectivity and historical patterns of diversification
Source: PeerJ. 2023 Jun 8;11:e15117. doi: 10.7717/peerj.15117 (PMC10257900; doi:10.7717/peerj.15117)

**Table S4.**  Pairwise *Φst* values for the mitochondrial COI marker, among Orinoco and Negro basin populations. Significantly different values (P<0.05) are shown in bold. Haplotype (h) and nucleotide (π) diversity ± standard deviation (SD) are shown on the diagonal for each geographic location. CAR: Puerto Carreño; GV: San José del Guaviare; IN: Puerto Inírida; PG: Puerto Gaitán; CUC: Cucui; SI: Santa Isabel; BAR: Barcelos.

| **Orinoco River basin** | | | | |  | **Negro River basin** | | |
| --- | --- | --- | --- | --- | --- | --- | --- | --- |
|  | **CAR** | **PG** | **GV** | **IN** |  | **CUC** | **SI** | **BAR** |
| **CAR** | ***h***= 0.4725 ±  0.135 | |  |  |  |  |  |  |
|  | ***π***= 0.3679 ±  0.002 | |  |  |  |  |  |  |
| **PG** | **0.8685** | ***h***= 0.2500 ±  0.180 | |  |  |  |  |  |
|  |  | ***π***= 0.1515 ±  0.001 | |  |  |  |  |  |
| **GV** | **0.7408** | 0.0559 | ***h***= 0.4000 ±  0.237 | |  |  |  |  |
|  |  |  | ***π***= 0.7345 ±  0.004 | |  |  |  |  |
| **IN** | **0.6738** | 0.1132 | 0.1150 | ***h***=0.5333 ±  0.180 | |  |  |  |
|  |  |  |  | ***π***= 1.0034 ±  0.005 | |  |  |  |
| **CUC** | **0.7895** | **0.9804** | **0.7659** | **0.5840** |  | ***h***= 0.0000 ±  0.000 | |  |
|  |  |  |  |  |  | ***π***= 0.0000 ±  0.000 | |  |
| **SI** | **0.7653** | **0.8452** | **0.6590** | **0.5931** |  | **0.7704** | ***h***= 0.6429 ±  0.184 | |
|  |  |  |  |  |  |  | ***π***= 0.6418 ±  0.004 | |
| **BAR** | **0.8517** | **0.9628** | **0.8347** | **0.7070** |  | **0.9551** | **0.6857** | ***h***= 0.2821 ±  0.141 |
|  |  |  |  |  |  |  |  | ***π***= 0.1548 ±  0.001 |

**Fig. S1.** Bayesian time-calibrated Maximum Credibility Tree, showing the phylogenetic reconstruction and ancestral relationship of Paracheirodn axelrodi, obtained from the spatiotemporal analysis by the Relaxed Random Walk method in BEAST v.1.8.2. Samples from Orinoco River basin [(a): Puerto Carreño; (b): Puerto Gaitán; (c): San José del Guaviare; (d): Inírida] and Negro River basin [(e): Cucuí; (f): São Gabriel da Cachoeira; (g): Santa Isabel; (h): Barcelos], using COI and Myh6 exonic region, estimated under a HKY+GAMMA and GTR+GAMMA models of molecular evolution, respectivelly. The observed times are median heights into 95% credibility intervals, calibrated with the widely accepted mtDNA substitution rate for poikilotherm vertebrates in Martin & Palumbi (1993) of 0.68x10-8 mutations per site per year for COI, and 1.0x10-9 for Myh6 (Freeland, 2005). Circles indicate nodes with supports ≥0.95 of posterior probability.


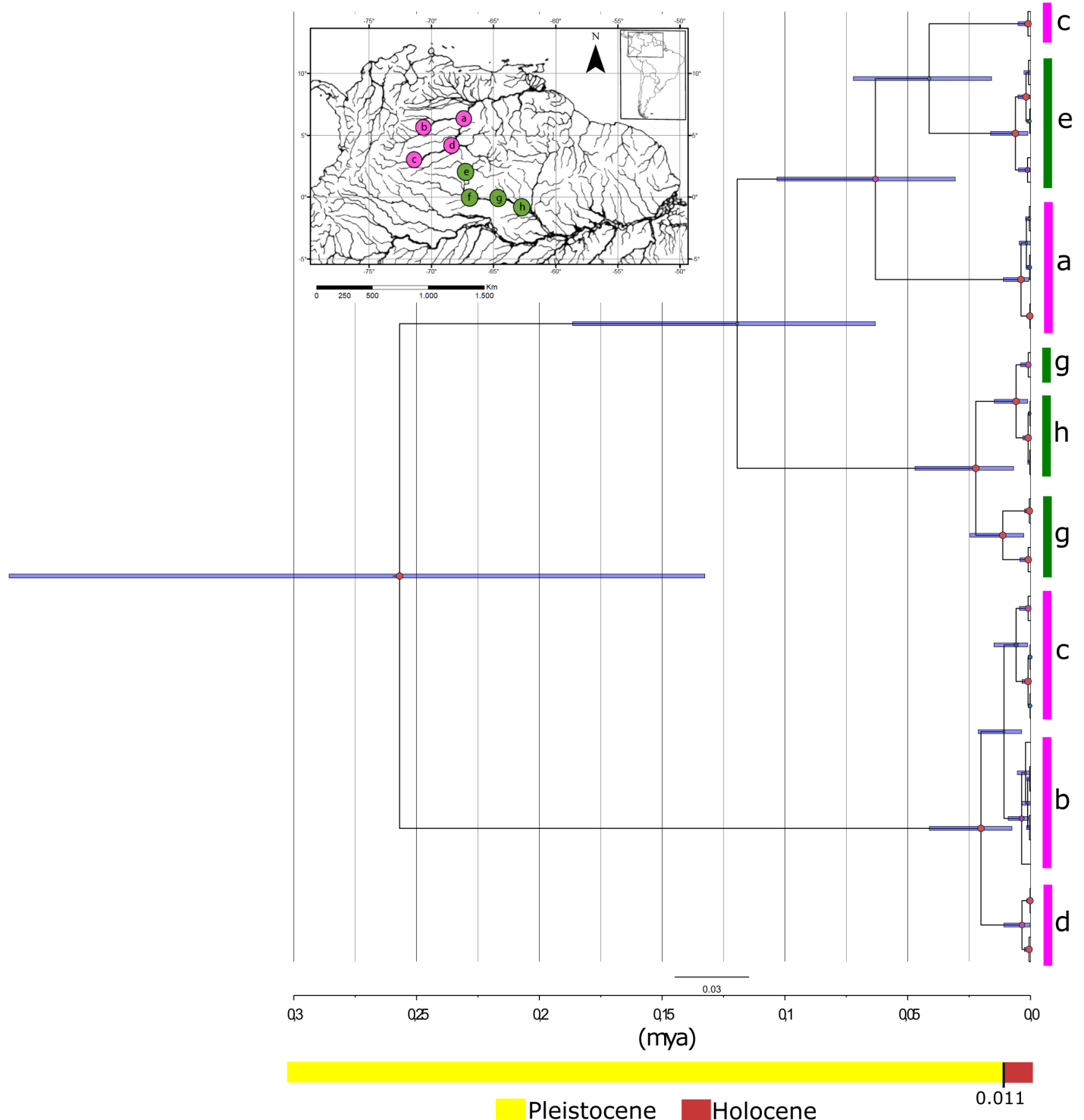

Supplement: Supplemental Information 11 — Significantly different values (P < 0.05) are shown in bold. Haplotype (h) and nucleotide (π) diversity ± standard deviation (SD) are shown on the diagonal for each geographic location. CAR, Puerto Carreño; GV, San José del Guaviare; IN, Puerto Inírida; PG, Puerto Gaitán; CUC, Cucuí; SI, Santa Isabel; BAR, Barcelos. [file peerj-11-15117-s011.docx]
